# The Effect of Warm-Up and Cool-Down Exercise on Delayed Onset Muscle Soreness in the Quadriceps Muscle: a Randomized Controlled Trial

**DOI:** 10.2478/v10078-012-0079-4

**Published:** 2012-12-30

**Authors:** Olav Olsen, Mona Sjøhaug, Mireille van Beekvelt, Paul Jarle Mork

**Affiliations:** 1 Department of Human Movement Science, Norwegian University of Science and Technology, Trondheim, Norway.

**Keywords:** aerobic exercise, pain, athletic injuries, pain threshold, muscles

## Abstract

The aim of the present study was to investigate the effect of warm-up and cool-down exercise on delayed onset of muscle soreness at the distal and central parts of rectus femoris following leg resistance exercise. Thirty-six volunteers (21 women, 15 men) were randomly assigned to the warm-up (20 min ergometer cycling prior to the resistance exercise), cool-down (20 min cycling after the resistance exercise), or control group performing resistance exercise only. The resistance exercise consisted of front lunges (10×5 repetitions/sets) with external loading of 40% (women) and 50% (men) of body mass. Primary outcomes were pressure pain threshold along rectus femoris and maximal isometric knee extension force. Data were recorded before the resistance exercise and on the two consecutive days. Pressure pain threshold at the central muscle belly was significantly reduced for the control group on both day 2 and 3 (p≤0.003) but not for the warm-up group (p≥0.21). For the cool-down group, pressure pain threshold at the central muscle belly was significantly reduced on day 2 (p≤0.005) and was also lower compared to the warm-up group (p=0.025). Force was significantly reduced on day 2 and 3 for all groups (p<0.001). This study indicates that aerobic warm-up exercise performed prior to resistance exercise may prevent muscle soreness at the central but not distal muscle regions, but it does not prevent loss of muscle force.

## Introduction

Delayed onset muscle soreness (DOMS) is common after intensive exercise and is particularly pronounced the days after unaccustomed eccentric muscle exercise (Cheung et al., 2003; Proske and Morgan, 2001). Eccentric contractions may induce temporary muscle damage (Proske and Morgan, 2001) followed by inflammation of the injured tissue (MacIntyre et al., 1996). Typically, pain and stiffness are not present until 8–24 hours after exercise and peak between 24 and 48 hours (Prasartwuth et al., 2005). Some studies indicate that muscle damage following eccentric exercise is associated with reduced muscle force capacity (Paulsen et al., 2010).

There is some evidence to support the notion that warm-up exercise prevents sports-related injuries (Fradkin et al., 2006). For example, aerobic warm-up exercise can be used to gradually speed up metabolic processes, elevate temperature in active muscles (Safran et al., 1989), and thereby increase muscle extensibility (Noonan et al., 1993). The duration of the warm-up exercise may appear important and it has been shown that at least 10–20 min of submaximal exercise is required for reaching a plateau in muscle temperature (Saltin et al., 1968). Thus, it may be hypothesized that a warm-up period with moderate aerobic exercise prior to intensive eccentric exercise will reduce DOMS by reducing the extent of muscle damage. Aerobic cool-down exercise may be an alternative method to reduce DOMS by increasing circulation and removal of noxious waste products in the exercised muscles (Cheung et al., 2003). However, studies investigating the effect of warm-up and/or cool-down exercise with an attempt to prevent or reduce DOMS have provided contradictory results (Takizawa et al., 2011; Gulick et al., 1996; Law and Herbert, 2007; Takahashi et al., 2006; Ingham et al., 2010; Evans et al., 2002; Takizawa et al., 2011). This inconsistency may be due to differences in exercise protocols and type of muscle work. Most studies investigated the preventive effect of the warm-up or cool-down in arm muscles, while focusing either on the effect of the warm-up or that of the cool-down. Gulick et al. (1996) investigated the effect of the warm-up by high-velocity concentric muscle contractions on DOMS in forearm extensor muscles and found no significant results. In contrast, Ingham et al. (2010) found a strong preventive effect of the warm-up by concentric elbow flexion on DOMS in elbow flexors. Furthermore, Evans et al. (2002) investigated the effect of the passive vs active warm-up on DOMS in elbow flexors and found a positive result of the passive warm-up. Finally, Takizawa et al. (2011) investigated whether 100 submaximal elbow flexion contractions (warm-up) would attenuate DOMS when performed prior to eccentric exercise. They found no effect of the warm-up, neither on DOMS or loss of muscle strength. Thus, none of these studies investigated the isolated effect of warm-up or cool-down exercise engaging larger muscle groups (i.e., leg muscles), which is relevant in many sport disciplines.

To our knowledge, only two studies investigated the preventive effect of the warm-up or cool-down in leg muscles, and only one of these studies looked at the warm-up and cool-down within the same study. Takahashi et al. (2006) investigated the effect of cool-down exercise (aqua exercise) after downhill running and found some significant effects, while Law and Herbert (2007) investigated the effect of 10 min uphill walking before (warm-up) and after (cool-down) walking backwards downhill on an inclined treadmill for 30 min and found some positive effect of the warm-up. Although these two studies investigated DOMS in the large muscle mass of the legs, the exercise intensity used for both warm-up and cool-down exercise was very low (i.e., 3.1–3.4 METs) and, therefore, possibly not optimal in creating a pronounced preventive effect on DOMS.

Moreover, none of the abovementioned studies investigated whether the effect of the warm-up or cool-down differed between intramuscular regions. A non-uniform distribution of muscle soreness after eccentric exercise has been reported for thigh muscles in previous studies (Baker et al., 1997; Hedayatpour et al., 2008). Since muscle fiber distribution and muscle oxygenation during exercise differ between intramuscular regions in anterior thigh muscles (Travnik et al., 1995; Kennedy et al., 2006), it is possible that warm-up and cool-down exercise impose different effects in different regions of the muscle.

The main objective of this study was to investigate the effect of aerobic warm-up and cool-down exercise on DOMS following intensive leg resistance exercise. A second objective was to investigate whether warm-up and cool-down exercise impose different effects on muscle soreness in different intramuscular regions.

## Material and Methods

### Participants

Thirty-six volunteers (21 women, 15 men) participated in the study. The inclusion criteria for the participants were that they were in good health, between 20 and 30 years of age, and practiced recreational physical exercise on a regular basis (1–4 exercise sessions per week), though should not compete in sports at international or national level. Exclusion criteria included 1) chronic diseases of the musculoskeletal system, 2) previous severe injuries to the back, hips, or knees, 3) current pain in back, hips, or knees, and 4) pregnancy. None of the participants had performed regular leg strength exercise in the previous 3 months. These criteria were created in order to avoid protection against DOMS from repeated bouts of resistance exercise. Eligible participants were randomly assigned into one of three groups; a warm-up group, a cool-down group, and a control group. Group characteristics at baseline according to group allocation are presented in Table 1. The allocation of participants was performed by random draw with men and women being assigned separately. The study was approved by the Regional Committee for Medical and Health Research Ethics (S-2009/1739-1, REK midt, Norway) and carried out in accordance with the Declaration of Helsinki.

### Measures and Procedures

Measurements were carried out on three consecutive weekdays with similar test time on each day (<2 hours difference between days). All participants performed a bout of front lunges on day 1. This resistance exercise imposes eccentric lengthening of the quadriceps muscle during the braking phase but also requires a concentric effort during the push-off phase. Precise and consistent description about the performance technique was given to each participant. The exercise was standardized by marking the individual stride length in the bottom position of the lunge when assuming a ∼90° angle in the knee and hip joint of the forward stepping leg. The exercise was performed with the dominant leg only, i.e., the forward stepping leg, in 5 sets with 10 repetitions with 30 sec rest between each set. A metronome was used to ensure participants maintained a cadence of 10 lunges per 30 sec. External load was provided by a barbell held behind the neck on top of the shoulders. The load was set to 40% and 50% of the body mass for woman and men, respectively.

Recordings of pressure pain threshold (PPT), maximal knee extension force during maximal voluntary isometric contraction (MVC), and subjective ratings of muscle soreness on a visual analogue scale (VAS) were carried out before the front lunge exercise (day 1), 24 hours after exercise (day 2), and 48 hours after exercise (day 3). All recordings were carried out for the exercised leg only. Prior to the front lunge exercise on day 1, the warm-up group completed 20 min of moderate intensity aerobic exercise. Conversely, for the cool-down group, the front lunge exercise was followed by 20 min of moderate intensity aerobic exercise. The control group only performed the front lunge exercise. The warm-up and cool-down were done on a cycle ergometer (Monark 939E, Vansbro, Sweden). The first 5 min of cycling was used to adjust the workload to correspond to ∼65% of estimated maximum heart rate (HR_max_ adjusted for age; 220-age * 0.65). The last 15 min was performed at a workload of 60–70% of HR_max_ with a cadence of 65–75 rpm. The choice of duration and workload was based on current recommendations for warm-up exercise (Bishop, 2003). The average workload (W) during the last 15 min was recorded for each participant. HR was recorded with a HR monitor (Polar RS800, Kempele, Finland). All participants were instructed to refrain from other vigorous physical activity during the experimental period.

A VAS consisting of a 100 mm line with end points; “no pain at all” (0 mm), and “worst pain imaginable” (100 mm) was used for subjective rating of muscle soreness. Participants were asked to rate the perceived pain intensity in the anterior thigh muscles of the exercised leg during ∼15 m walking at preferred speed, i.e., walking without hurrying. PPT was measured sequentially at six locations along the rectus femoris muscle in the exercised leg using a hand held electronic pressure algometry with a probe area of 1cm^2^ (Somedic Algometer Type II, Sweden). The locations corresponded to 10, 20, 30, 40, 50, and 60% of the distance from the superior patella border to the anterior superior iliac spine. A similar approach to record PPT along the rectus femoris had been described previously in the study of Hedayatpour et al. (2008). The locations were marked to ensure that PPT was recorded at the same locations on all three days. PPT recordings were carried out in a seated position with a ∼90° angle in the hip and knee joint and performed twice at each location on each day, starting with the most distal point and ending with the most proximal point for both recordings. The mean PPT of the two recordings at each location was used in further analysis.

During PPT recordings, the algometry was applied perpendicular to the skin with an application rate of 40 kPa/sec. The participants were holding a stop button connected to the algometry and were instructed to press the button when the sensation of pressure changed to pain. Pressing down the button instantly froze the reading and the downward pressure was ceased immediately. In further analysis, three different PPT variables were derived; 1) mean PPT of the 10–60% locations, denoted as PPT_distal+central_, 2) mean PPT of the 50 and 60% locations, denoted as PPT_central_, and 3) mean PPT of the 10 and 20% locations, denoted as PPT_distal_. PPT_distal_ is assumed to primarily reflect PPT at the muscle part close to the myotendinous junction while PPT_central_ mainly provides a measure of PPT at the mid-belly of the muscle.

Maximal isometric knee extension force during MVC was recorded three times for the exercised leg with 1 min rest between recordings. Participants were instructed to build up maximal force within 1–2 sec and then hold that force for 4–5 sec. Force was measured by a force transducer (SM-2000N, Interface MFG, Scottsdale, USA) and recorded by a Delsys bagnoli-16 system (Delsys Inc., Boston, USA). The force signal was low-pass filtered (Butterworth, 10 Hz, 6^th^ order) and down sampled to 0.1 sec time resolution before further analysis. The force transducer was embedded in a belt strap which was fixed around the participant’s ankle enclosing their lateral malleolus and secured to the base of an adjustable chair. Force recordings were carried out with the participant in a seated position with Velcro belts fastened across the chest and waist with knee and hip angles at ∼90°. The average of the maximal force output during the three MVCs was determined and used in further analysis.

### Statistical Analysis

SPSS for Windows (version 17.0) was used for all statistical analyses. One-way ANOVA was used to investigate baseline differences between groups. A mixed design ANOVA (3x3) was used to investigate changes in force and PPT with one between-subjects variable, *group*, with three levels (warm-up, cool-down, control) and one within-subject variable, *time*, with three levels (day 1–3). The effect of group, time, and group by time interactions were tested. When the assumption of sphericity was violated, significance was adjusted using the Greenhouse-Geisser method. When the effect of time was significant, a Bonferroni post-hoc test was used to investigate within-group differences. An independent samples *t*-test was used to investigate between-group differences at day 2 and 3 when the effect of group was significant. All PPT variables showed a non-normal distribution (positively skewed). Logarithmic transformed data was therefore used in the statistical analysis.

VAS ratings of muscle soreness were analyzed using non-parametric tests due to non-normal distribution of the data (negatively skewed). One-way ANOVA by ranks (Kruskal-Wallis) was used to investigate group differences within days while the Friedmans’s test was used to investigate differences between days. The Wilcoxon signed-rank test was used for pairwise comparisons. Statistical significance was set at p<0.05 for all comparisons.

## Results

There were no group differences for age, body mass index, PPT, or knee extension force at baseline (Table 1). Neither was there any difference in the average workload during the cycling exercise between the warm-up and cool-down group (61±20 W vs. 60±22 W, p=0.92).

**Figure 1:**
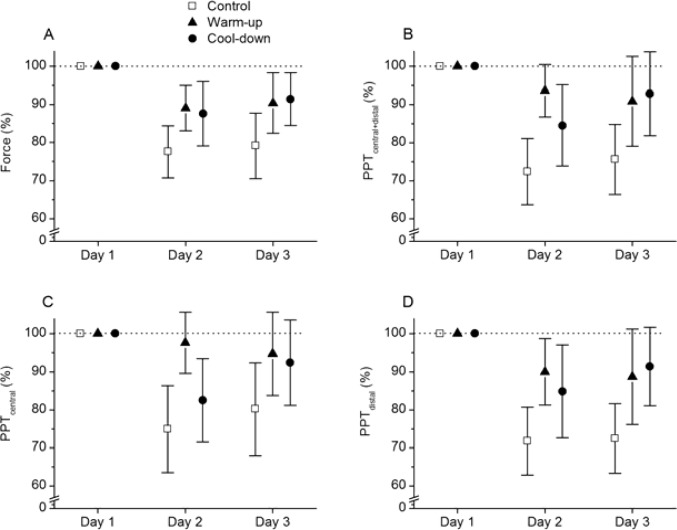
Relative values for maximal isometric knee extension force (A), PPT_central+distal_ (B), PPT_central_ (C), and PPT_distal_ (D) before (day 1), and after (day 2 and 3) intensive leg resistance exercise. Baseline values (day 1) are presented as 100% indicated by the horizontal dotted line. Group belonging is indicated by symbols and error bars indicate 95% confidence interval of mean.

Figure 1 presents relative values for maximal isometric knee extension force during MVC (A), PPT_central+distal_ (B), PPT_central_ (C), and PPT_distal_ (D) on day 1–3. Baseline values (day 1) are presented as 100% indicated by dotted horizontal lines. There was a significant main effect of time on force (F=31.01, p<0.001) but no group by time interaction (F=2.14, p=0.087). Post-hoc tests showed that the main effect of time reflected significantly lower force on both day 2 and day 3 compared to day 1 (p<0.001 for all within-subject comparisons).

There was a significant difference of time in PPT_central+distal_ (F=19.68, p<0.001), PPT_central_ (F=15.78, p<0.001), and PPT_distal_ (F=22.32, p<0.001). With regard to the group by time interaction, a significant difference was found for PPT_central+distal_ (F=2.83, p=0.031) and PPT_central_ (F=2.93, p=0.027), but not for PPT_distal_ (F=2.26, p=0.072). As shown in Figure 1, this indicates that PPT_central+distal_ and PPT_central_ decreased more on day 2 and 3 within the control group than within the experimental groups.

For the control group, post-hoc tests revealed that PPT_central+distal_ was significantly lower on day 2 (p<0.001) and day 3 (p<0.001) compared to day 1. In contrast, no significant change was found in PPT_central+distal_ for the warm-up group, neither from day 1 to day 2 (p=0.57), nor from day 1 to day 3 (p=0.21).

The cool-down group showed a significant reduction in PPT_central+distal_ from day 1 to day 2 (p=0.005) but no significant reduction for PPT_central+distal_ from day 1 to day 3 (p=0.38). The warm-up group tended to have higher PPT_central+distal_ on day 2 compared to the cool-down group. However, this comparison did not reach significance (p=0.087). Similar findings were made for PPT_central_ as for PPT_central+distal_. For the control group, PPT_central_ was significantly lower on day 2 (p<0.001) and day 3 (p=0.003) compared to day 1 while PPT_central_ did not change for the warm-up group, neither from day 1 to day 2 (p=1.0), nor from day 1 to day 3 (p=0.74). The cool-down group had a significant reduction in PPT_central_ from day 1 to day 2 (p=0.004) but not from day 1 to day 3 (p=0.25). Group comparisons showed that PPT_central_ was higher for the warm-up group than the cool-down group on day 2 (p=0.025) but not on day 3 (p=0.09).

**Figure 2:**
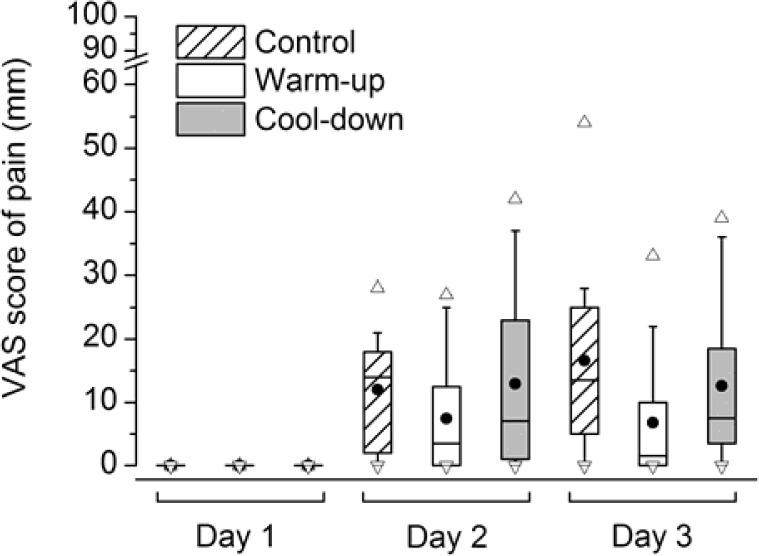
Box-plot of pain scores on visual analogue scale (VAS) before (day 1) and after (day 2 and 3) intensive leg resistance exercise. Boxes include the 25–75 percentiles and whiskers include 10–90 percentiles. Mean is indicated by black circles and median by horizontal line within boxes. Outliers are indicated by open triangles.

Figure 2 presents VAS scores for pain in the anterior thigh muscles of the exercised leg. All subjects scored zero on VAS ratings of muscle soreness on day 1 and there was a significant effect of time within all groups (p<0.006 for all within-subject comparisons). The median VAS rating within the control group was 14 mm (range 0–28) on day 2 and 14 mm (range 0–54) on day 3. The corresponding VAS ratings were 4 mm (range 0–27) and 2 mm (range 0–33) within the warm-up group and 7 mm (range 0–42) and 8 mm (range 0–39) within the cool-down group. There were no significant differences between groups in VAS ratings, neither on day 2 (p=0.46) nor on day 3 (p=0.12).

## Discussion

The current study indicates that the aerobic warm-up prior to resistance exercise to some extent prevents muscle soreness at the central muscle belly. A significant group by time interaction was observed for the overall PPT (PPT_cental+distal_) as well as for PPT at the mid-belly (PPT_central_) of rectus femoris, indicating that these measures of PPT changed differently with time in the three groups. In contrast to within the control group, no significant reduction was observed in PPT_central+distal_ or PPT_central_ within the warm-up group from baseline to 24 hours (day 2) or 48 hours post-exercise (day 3). The reduction of PPT was substantially larger for the control group than for the warm-up group, especially for PPT_central_ (∼20–25% reduction in the control group versus ∼3–5% reduction in the warm-up group). Moreover, PPT_central_ was significantly higher for the warm-up group than the cool-down group on day 2 and also tended to be higher on day 3. This may indicate that aerobic warm-up exercise is more effective in preventing muscle soreness the first 24 hours compared to aerobic cool-down exercise. However, the finding of less reduction of PPT in the experimental groups compared to the control group was not reflected by the subjective VAS ratings of perceived pain in the anterior thigh muscles. Both experimental groups and the control group reported higher VAS ratings on day 2 and day 3 compared to day 1 but VAS ratings did not differ between groups.

Moderate intensity cycling was chosen as warm-up and cool-down exercise for several reasons. First, cycling activates large muscle groups that leads to a relatively steep rise in muscle temperature that reaches a plateau after 10–20 min (Saltin et al., 1968). Increased muscle temperature may increase muscle extensibility (Noonan et al., 1993), thereby reducing the risk of muscle damage from overstretched muscle fibers. Second, cycling mainly constitutes concentric muscle work (Ettema and Loras, 2009). Other common types of exercise that activate the leg muscles such as jogging/running induce eccentric tension of the leg extensor muscles when the downward motion is decelerated after the foot touches the ground. This eccentric activation may result in muscle damage (Armstrong et al., 1983). Exercise that mainly constitutes concentric muscle contractions may therefore be a better choice for the warm-up and cool-down before and after leg resistance exercise to avoid aggravation of muscle damage by additional eccentric contractions. This may be particularly relevant for the cool-down exercise and there is some evidence indicating that concentric cool-down exercise performed in water results in enhanced recovery of flexibility of the leg muscles compared to jogging (Takahashi et al., 2000).

Similar to our findings, previous studies have indicated that warm-up exercise prior to eccentric exercise is associated with less reduction of PPT in lower (Law and Herbert, 2007) and upper limb muscles (Ingham et al., 2010). One study included cool-down exercise but found no effect on PPT after eccentric exercise (Law and Herbert, 2007). However, none of these studies investigated whether warm-up or cool-down exercise had a different effect on PPT at different intramuscular regions. A non-uniform distribution of PPT along the rectus femoris after eccentric exercise has been reported in previous studies (Baker et al., 1997; Hedayatpour et al., 2008). However, while Baker et al. (1997) found the largest reduction in PPT at the central region, Hedayatpour et al. (2008) found it at the distal region. The reason for these contrasting findings is unclear but may relate to different protocols for inducing DOMS, i.e., downhill running (Baker et al., 1997) versus leg exercise in a dynamometer (Hedayatpour et al., 2008). In our study, the similar relative reduction of PPT at the distal and central regions within the control group (i.e., ∼20–30% at both sites) may indicate that the muscle damage was of similar magnitude in both regions. Thus, the site-specific differences in PPT between the control group and the experimental groups at day 2 and day 3 are likely caused by site-specific responses to the intervention.

A possible explanation for the differential effect at the central and distal muscle region may relate to a graded vascularization throughout the muscle belly. Compared to the mid-belly of quadriceps a higher density of fast-twitch fibers was found in the distal region of this muscle (Travnik et al., 1995). Fast-twitch fibers have less capillary-to-fiber ratio than slow-twitch fibers (Sjøgaard, 1982) and muscle regions with relatively high density of fast-twitch fibers may therefore have a less advantageous effect of aerobic warm-up and cool-down exercise. This notion is partly supported by a study showing less muscle oxygenation in the distal versus central muscle region in vastus lateralis during exercise (Kennedy et al., 2006). Moreover, fast-twitch fibers may be more susceptible to damage following eccentric exercise compared to slow-twitch fibers (Takekura et al., 2001). It should also be noted that the warm-up and cool-down exercise in the current study was performed with low force concentric muscle work that will mainly activate slow-twitch fibers. Thus, our findings may suggest that the preventive effect of moderate intensity warm-up and cool-down exercise on hyperalgesia is more pronounced in muscle regions or muscles that are well vascularized with high density of slow-twitch fibers. However, this notion lacks empirical support and needs to be confirmed in future studies.

Although the average VAS ratings of muscle soreness in the anterior thigh muscles were more than threefold higher in the control group than in the warm-up group on both day 2 and day 3, the difference did not reach significance due to the large inter-individual variation in pain scores. In this study, the participants were instructed to rate muscle soreness during walking a short distance at preferred speed and the pain scores were quite low. Typically, the perceived muscle pain associated with DOMS is more intense during forceful contractions of the affected muscle. It is therefore possible that the difference between the control group and the experimental groups would have been more distinct if VAS ratings were obtained during more forceful contractions of the quadriceps muscle. Moreover, the lack of difference between the control group and the experimental groups may be due to the convergence of nociceptive input from other regions of the quadriceps muscle to the dorsal horn of the spinal cord (Kosek and Hansson, 2003). Thus, the perceived muscle pain is likely to be dominated by the most intensive input (i.e., similar mechanism as for referred pain), which in this case could originate from any part of the anterior thigh muscles.

A prolonged force decline after eccentric exercise is considered a valid and reliable indirect marker of muscle damage (Warren et al., 1999). Previous studies have provided contradictory results regarding the preventive effect of the warm-up or cool-down on force decline (Gulick et al., 1996; Ingham et al., 2010; Takahashi et al., 2006). However, the exercise protocols vary considerably between different studies and none of them investigated the effect of aerobic warm-up or cool-down exercise. We observed a tendency for a more pronounced force decline in the control group (∼20% decline, day 2 and 3) compared to both experimental groups (∼10–12% decline, day 2 and 3); however, due to our limited sample size these differences did not reach significance. Thus, based on the mixed results in our and previous studies, it is difficult to draw any firm conclusion regarding the effect of aerobic warm-up and cool-down exercise on force decline after eccentric exercise.

There are several strengths of our study, such as the comparison of randomized groups, no withdrawals or missing data, and the outcome variables specified *a priori*. PPTs were recorded by a force transducer algometer which is a well-established and highly reliable method for measurement of hyperalgesia (Jones et al., 2007). Likewise, recordings of acute pain by VAS (Bijur et al., 2001) and recordings of isometric knee extension force have been shown to be highly reliable (Sole et al., 2007). Moreover, we used an identical protocol for the warm-up and cool-down exercise which enabled a direct comparison of the effect of these two exercise modalities on DOMS following resistance exercise. However, it is possible that the warm-up and cool-down have an additive effect on prevention of DOMS and this should be investigated in future studies. Previous experience with physical exercise and leg strength may also influence on the effect of warm-up and cool-down exercise. Although no data is available for women, leg strength of our male participants corresponded well with leg extension torque recorded in other studies of young healthy male adults. The converted torque values for our male subjects (n=15; 222 ± 25.3 Nm) were in agreement with the knee extension torque of 231 ± 9 Nm reported by Thorstensson et al. (1976) and the 10th (∼160 Nm) and 90th (∼275 Nm) percentile reported by Alangari and Al-Hazzaa (2004). None of our participants was competing at the national or international level, but all participated in recreational physical activity on a regular basis. Whether our findings can be generalized to individuals with either higher or lower physical capacity, e.g., elite athletes or patients groups, remains uncertain. It should also be noted that PPT was only recorded from the rectus femoris muscle and we cannot conclude whether the warm-up and cool-down have a general effect on the quadriceps muscle. Finally, a longer follow-up period until all variables returned to baseline would have enabled a more thorough characterization of the recovery pattern.

In conclusion, it may be stated that the present study indicates that aerobic warm-up exercise of moderate intensity with mainly concentric muscle work performed prior to intensive leg resistance exercise may prevent muscle soreness but not loss of muscle force. The preventive effect on muscle soreness is most pronounced at the central muscle region. Cool-down exercise may be less effective than the warm-up in attenuating muscle soreness the first 24 hours after resistance exercise. The practical implication is that moderate aerobic warm-up or cool-down exercise, with mainly concentric muscle work, can be recommended to attenuate muscle soreness following intensive leg resistance exercise.

## Figures and Tables

**Table 1:** Group characteristics at baseline according to group allocation.

	Groups			
	Control	Warm-up	Cool-down	p*
Male/female	5/7	5/7	5/7	N/A
Age (years)	23±3	23±3	22±3	0.91
Body mass index (kg/m^2^)	23.8±1.9	23.2±1.8	24.0±4.8	0.81
PPT_central_ (kPa)	689±401	769±305	566±216	0.30
PPT_distal_ (kPa)	891±430	964±445	760±329	0.47
PPT_central+distal_ (kPa)	788±406	861±365	660±261	0.37
Max. knee extension force (N)	455±162	503±107	508±107	0.56

Values are mean±SD unless otherwise stated.

Abbreviations: N/A, not applicable; PPT, pressure pain threshold.

* p-value of one-way ANOVA
